# Beginning a New Year

**Published:** 1916-07

**Authors:** 


### EDITORIAL DEPARTMENT

MRS. F. E. DANIEL, Publisher and Managing Editor.

### Beginning a New Year.

With this issue the TEXAS MEDICAL JOURNAL enters upon its thirty-second year. Thirty-one years is a ripe age for publications of any kind, and is generally considered old enough to insure their continuance. Some publishers make the mistake of laying energy aside when their publications begin to attain age enough to command business; but to relax effort because of age, or of circulation, or of influence, always proves fatal, and sooner or later brings failure. But given the experience of age and the vigor of youth along with the prudence and judgment that should characterize maturity, publications should be better off for each added year.

It is hoped that the readers and patrons of the TEXAS MEDICAL JOURNAL may not think a reference to its past achievements is arrogant self-praise. From the very beginning, when the JOURNAL was established by Dr. F. E. Daniel, it commanded the respect of all thinking people in the profession. It was recognized as a power of strength by those who were progressive in their studies and who were anxious to see medicine and surgery and the kindred subjects keep pace with the progress of the world. When Dr. Daniel, a little more than two years ago, passed to his reward, his work was taken up by the present publisher with many doubts and misgivings, but not entirely without preparation and experience. Friends in the profession, who must have shared in the publisher's fears, rallied nobly to the support of the JOURNAL, volunteering in many cases their assistance. To that loyal support, rather than to any personal merit, is the success of the publication due in greatest part. For this great assistance thanks are here given, and it is hoped that every person who has by contribution of articles, by advertising or subscription patronage, by letters and words of encouragement lessened the writer's burdens and made the work easier, may regard this as a personal acknowledgment of even the smallest favor. Certainly every bit of assistance has been greatly appreciated, and the publisher wishes it were possible to give personal thanks to the thousands who have been so kind.

But there is nothing to be gained by looking at the past, except as the retrospect may cause greater and better effort for the future. The success of the past two years—thanks again to the helpful interest of friends—has far exceeded all anticipations, but it has created an intense desire to do still bigger and better things, to make the TEXAS MEDICAL JOURNAL so good that it will become a welcome visitor in every physician's office in the South, which is the field it especially tries to cover. Plans have been made to bring that about, and the help of a number of the best medical men of the country has been promised. Announcement has already been made of some of the things now being undertaken, and elsewhere in this number other good things in store are mentioned. These are but a part of the general plan for the improvement of the JOURNAL, announcement of which will be made from time to time. Suffice it to say now that nothing will be left undone that experience, energy, effort and wise counsel of good friends can suggest to make the TEXAS MEDICAL JOURNAL equal to any professional journal of this country in all respects and superior in many. Already several of its features are the best to be found anywhere and to these additions are rapidly being made. Appreciating this, the physicians of the country are responding with their subscriptions, nearly one thousand having been added in the past two months. Is it too much to expect to increase that number ten fold within the next six months? That is the goal toward which the TEXAS MEDICAL JOURNAL is striving. To do this, it asks your continued co-operation, here making the promise on its birthday that it will do everything in its power to fully merit your assistance.

